# Diagnosis and treatment of sarcoidosis based on immunological etiology and mechanisms

**DOI:** 10.3389/fmed.2026.1754491

**Published:** 2026-02-12

**Authors:** Jingshu Zhao, Lixuan Ma, Junwen Luan

**Affiliations:** 1 Department of Pathogen Biology, School of Clinical and Basic Medical Sciences, Shandong First Medical University & Shandong Academy of Medical Sciences, Jinan, Shandong, China; 2 Department of Clinical Laboratory Medicine, The First Affiliated Hospital of Shandong First Medical University & Shandong Provincial Qianfoshan Hospital, Jinan, Shandong, China

**Keywords:** biomarkers, immune mechanisms, pathogenesis, pulmonary sarcoidosis, step-up therapy

## Abstract

Pulmonary sarcoidosis is a multisystem disease characterized by non-caseating granulomas, and its pathogenesis involves genetic susceptibility and abnormal immune responses triggered by environmental antigens. This article reviews the immunological etiology and mechanisms of pulmonary sarcoidosis, systematically discussing its pathogenetic basis, core immune mechanisms, and the role of key immunological biomarkers (such as sIL-2R, CD4+/CD8+ ratio in BALF, and HRCT) in diagnosis and assessment. It also outlines the step-up therapy, ranging from glucocorticoids to immunomodulators, biologic agents, and emerging anti-fibrotic therapies, aiming to provide an immunological basis for the precise diagnosis and treatment of pulmonary sarcoidosis.

## 1 Introduction

Sarcoidosis is a disease characterized by non-caseating granulomas and can involve almost all organs of the body (1). Studies indicate that over 90% of sarcoidosis patients have pulmonary involvement (2), and 60% of patients die from this cause (3). Currently, it is believed that specific microbial antigens can serve as triggers for sarcoidosis in genetically susceptible individuals. Supporting this, *Propionibacterium acnes* has been localized within sarcoid granulomas, suggesting a direct etiologic role (4). Its essence is an abnormal Th1-type immune response, but traditional diagnosis and treatment are mostly based on clinical manifestations, lacking specificity with respect to the etiology and immune mechanisms (5–7). With the advancement of immunological research, approaching from the perspective of immuno-etiological mechanisms can provide new perspectives and strategies for the precise diagnosis and effective treatment of pulmonary sarcoidosis. This article aims to systematically review the etiology, diagnostic biomarkers, and treatment strategies of pulmonary sarcoidosis from an immunological standpoint, intending to provide a theoretical foundation and practical guidance for clinical practice.

## 2 Immunological etiology and mechanisms of pulmonary sarcoidosis

The onset of pulmonary sarcoidosis begins with an abnormal immune reaction triggered by environmental antigens in genetically susceptible individuals.

### 2.1 Pathogenetic basis

Genetic susceptibility is the foundation for disease development. Specific HLA alleles (such as HLA-DRB1*03, *07, DRB3/4/5, DPB1, DPA1, DQA1) are significantly associated with different clinical manifestations and prognoses (8). Polymorphisms in non-HLA genes (e.g., *BTNL2*, *TNF-*α genes) also elevate disease risk (9, 10). Environmental exposure is a critical trigger. Inhaled agents such as metal dusts, silica dust (11), and microbial antigens (e.g., *Propionibacterium acnes*, mycobacterial components) are considered potential antigen sources, which may disrupt immune tolerance through molecular mimicry or persistent antigen stimulation (12). Additionally, immune checkpoint inhibitors can also induce sarcoid-like reactions (13).

### 2.2 Core immunological mechanisms

The classical immunological mechanism involves the polarization of CD4+ T cells into Th1/Th17 cells, which secrete large quantities of key cytokines such as IFN-γ, TNF-α, and IL-17, driving macrophage activation and the formation of non-necrotizing granulomas (5). Recent studies have significantly deepened this understanding. On the innate immunity front, metabolic reprogramming in macrophages–particularly the activation of the mTORC1–HIF-1α axis–has been established as a core driver of granuloma formation and maintenance (14, 15). In adaptive immunity, the research focus has shifted from classical Th1 cells to Th17.1 cells, which are recognized as the primary source of IFN-γ in the pulmonary microenvironment and are closely associated with disease chronicity and glucocorticoid resistance (16). Furthermore, B cells participate in *in situ* immune responses through the formation of tertiary lymphoid structures, revealing a new dimension of localized adaptive immune activation (17). The chronic nature of the disease and its progression to fibrosis are closely related to regulatory T cell (Treg) dysfunction and the persistent activation of pro-inflammatory signaling pathways (e.g., the OX40/OX40L pathway) (18, 19).

## 3 Diagnostic strategies based on immuno-etiological mechanisms

### 3.1 Key immunological biomarkers

The diagnosis of pulmonary sarcoidosis requires a combination of clinical, imaging, and pathological findings, but assessing its immune activity is crucial for clinical decision-making, treatment evaluation, and prognosis. The core of this diagnostic strategy is the use of specific biomarkers to evaluate the immune status. Over the past decades, serum angiotensin-converting enzyme (SACE) has been routinely used in the clinical management of sarcoidosis. It is secreted by monocytes, macrophages, and epithelioid cells, and contributes to the regulation of granuloma formation in sarcoidosis. However, given its relatively low sensitivity and high specificity, SACE alone appears insufficient to establish a diagnosis of sarcoidosis based solely on this biomarker (20). In serological testing, the serum soluble IL-2 receptor (sIL-2R), serving as a quantitative marker of T-cell activation with sensitivity superior to SACE, is the preferred serological indicator for monitoring treatment response and predicting relapse (21). For the assessment of the local immune microenvironment, bronchoalveolar lavage fluid (BALF) immunophenotyping is essential; notably, a CD4+/CD8+ ratio > 3.5 is highly suggestive of sarcoidosis (specificity 93%–96%), directly reflects a local Th1 immune bias in the lungs, and constitutes important evidence supporting the diagnosis, while the expression of activated T-cell markers (such as CD38) aids in differentiation from other interstitial lung diseases (22). Key differentiating features is shown in Table 1.

**TABLE 1:** The role of BALF analysis in the differential diagnosis of sarcoidosis.

Disease category	Key BALF^a^ immunophenotype	Diagnostic implication
Sarcoidosis	CD4+/CD8+ ratio > 3.5 (high specificity)	Strongly supports diagnosis
Tuberculosis	CD4+/CD8+ ratio < 1.0	Differentiates from sarcoidosis
IPF^b^/fibrotic ILD^c^	Not characteristic	Suggests non-granulomatous process
Other ILD (e.g., HP^d^)	CD4+/CD8+ ratio low/normal (<2.0)	Indicates CD8+ predominance
Berylliosis	CD4+/CD8+ ratio can be elevated	Diagnosis requires exposure history and BeLPT^e^

This table synthesizes key findings from El Fakihi et al. (22). The CD4+/CD8+ ratio is a central discriminator: a high ratio (>3.5) strongly favors sarcoidosis, while a low ratio (<1.0) typically suggests alternative etiologies such as infection or HP.

^a^BALF, bronchoalveolar lavage fluid.

^b^IPF, idiopathic pulmonary fibrosis.

^c^ILD: interstitial lung disease.

^d^HP, hypersensitivity pneumonitis.

^e^BeLPT, beryllium lymphocyte proliferation test.

In recent years, chitotriosidase (CHIT), a novel serum biomarker secreted by activated macrophages, has shown promising value in the diagnosis of sarcoidosis. Its activity is significantly elevated in patients, with diagnostic sensitivity and specificity exceeding 86% and 93%, respectively (23). Furthermore, CHIT levels decrease following treatment, supporting its utility in monitoring therapeutic efficacy (23). CHIT demonstrates superior diagnostic performance compared to the conventional biomarker angiotensin-converting enzyme (ACE). Notably, the “double product” (ACE × CHIT), derived from the multiplication of both markers, further enhances diagnostic accuracy and outperforms either biomarker alone (24). Additionally, the diagnostic efficacy of CHIT is comparable to that of BALF CD4+/CD8+ ratio, offering a reliable non-invasive assessment tool (25).

### 3.2 Imaging diagnosis and its immunological significance

Imaging plays a pivotal role in the diagnosis, staging, and assessment of pulmonary sarcoidosis. Chest X-ray typically shows symmetrically enlarged bilateral hilar lymph nodes, with or without pulmonary infiltrates, serving as a common tool for initial screening and staging. High-resolution computed tomography (HRCT) is the cornerstone of imaging evaluation, providing superior visualization of subtle changes in lymph nodes and lung parenchyma. Typical HRCT findings include symmetrically enlarged bilateral hilar and mediastinal lymph nodes. On contrast-enhanced CT, these nodes frequently demonstrate moderate to marked homogeneous enhancement without fusion, aiding in differentiation from conditions such as lymphoma (which may show mild enhancement) or tuberculosis (which can present with rim enhancement) (26, 27). Furthermore, HRCT can reveal intrapulmonary micronodules distributed along lymphatic pathways, reticulation, traction bronchiectasis, and other fibrotic changes, offering significant value in assessing disease activity and the extent of fibrosis.

**FIGURE 1:**
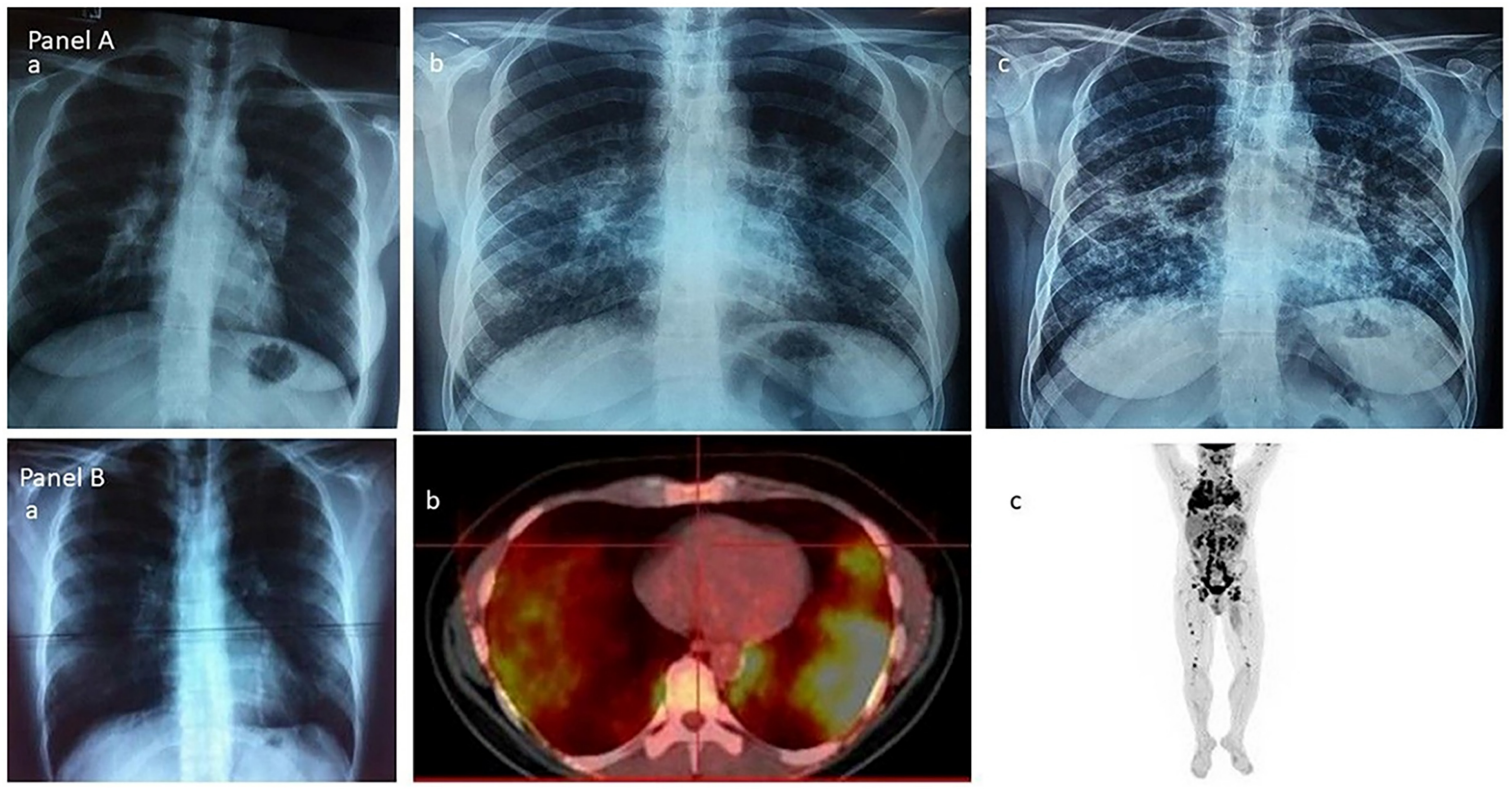
**(A)** The Scadding’s vision of sarcoidosis through the chest roentgenogram: group 1–enlarged hilar lymph-nodes; group 2–hilar nodes and lung shadowing; and group 3–lung shadowing. **(B)** The current vision of sarcoidosis through the 18F-FDG PET/CT scan: (a) and (b) the lungs; (c) the whole body. Adapted from Papiris et al. (28) under a CC-BY4.0 license.

Furthermore, FDG-PET/CT functional imaging non-invasively assesses granulomatous metabolic activity and is of significant diagnostic value for pulmonary sarcoidosis (28). The images of chest X-ray and 18F-FDG PET/CT chest X-ray are shown in Figure 1. This technique is particularly valuable in clinical practice for several key applications. Primarily, it serves to accurately evaluate systemic disease activity and monitor treatment response. A reduction in the maximum standardized uptake value (SUVmax) post-treatment strongly correlates with improvements in pulmonary function parameters, such as forced vital capacity (FVC) and diffusing capacity for carbon monoxide (DLCO) (29). Secondly, PET/CT offers unique advantages in detecting occult extrapulmonary involvement (e.g., cardiac, neural, or bone marrow), thereby aiding in comprehensive staging (30). Thirdly, it can guide biopsy site selection by identifying the most metabolically active lesions, thereby increasing diagnostic yield (31). It is therefore mainly recommended for cases with diagnostic uncertainty, suspected multi-organ involvement, or treatment refractoriness.

A meta-analysis provides high-level evidence for its diagnostic performance: the pooled sensitivity reached 0.971 (95% CI 0.909–1.000), meaning this technique can identify 97.1% of true cases with a very low missed diagnosis rate; the pooled specificity was 0.873 (95% CI 0.845–0.920), indicating it can accurately exclude 87.3% of non-sarcoidosis lesions, demonstrating good differential diagnostic capability (30). Notably, FDG-PET/CT is advancing from qualitative assessment to quantitative prognostic prediction. Volume-based metabolic parameters, such as metabolic tumor volume (MTV) and total lesion glycolysis (TLG), comprehensively quantify the systemic granulomatous burden. Higher baseline MTV or TLG independently correlates with accelerated lung function decline and increased disease progression risk (32), providing a key tool for risk stratification and personalized management in pulmonary sarcoidosis.

### 3.3 24 -hour urinary calcium monitoring

The measurement of 24-h urinary calcium has transcended its traditional role in monitoring calcium metabolism in pulmonary sarcoidosis, emerging as a significant biomarker that reflects systemic granulomatous immune activity and aids in the differentiation of fibrotic phenotypes. Elevated levels correlate positively with macrophage activation markers (e.g., chitotriosidase) and are associated with a decline in diffusing capacity, indicating its utility in assessing disease activity and severity (33, 34). Crucially, in the challenging differential diagnosis between stage IV fibrotic sarcoidosis and idiopathic pulmonary fibrosis or chronic hypersensitivity pneumonitis, 24-h urinary calcium demonstrates high specificity (approximately 89.7%) and good discriminatory power (AUC 0.77), outperforming serum calcium (35). Therefore, this simple, non-invasive test provides valuable evidence for the precise diagnosis and evaluation of sarcoidosis, particularly its fibrotic subtype.

### 3.4 The significance of fundus examination in screening uveitis

Uveitis is a common and important extrapulmonary manifestation of pulmonary sarcoidosis. Comprehensive fundus examination holds critical diagnostic value in screening. Clinical practice has confirmed that systemic inflammation in sarcoidosis can directly coincide with or accompany uveitis. For example, Kawali et al. reported a case of biopsy-confirmed pulmonary sarcoidosis that was concurrently diagnosed with Fuchs uveitis presenting atypical signs during the disease course (36). This suggests that systematic ophthalmic screening is essential for sarcoidosis patients to promptly detect granulomatous inflammation or specific comorbidities. Additionally, another case demonstrated that a patient initially suspected of tuberculosis was found to have optic disk granuloma and periphlebitis upon fundus examination, ultimately leading to a revised diagnosis of systemic sarcoidosis (37). This further illustrates the indicative role of fundus findings in systemic diagnosis and their value in differential diagnosis. These fundus features are also included in the International Workshop on Ocular Sarcoidosis (IWOS) diagnostic criteria (38).

### 3.5 Etiology-based antigen detection

In addition to assessing immune activity, directly identifying antigens is a key advancement toward etiological diagnosis. For example, specific immunohistochemical staining for *Propionibacterium acnes* (using PAB antibody) can visualize this microorganism within granulomas and help distinguish sarcoidosis from other granulomatous diseases (39). This immunohistochemical approach provides a potential method for disease subtyping from an etiological perspective, supplementing immune biomarkers. It should be noted, however, that this finding originates from a relatively small-scale study, and the technique has not yet been incorporated into current diagnostic guidelines, remaining a primarily research tool.

## 4 Step-up and targeted therapy based on immuno-etiological mechanisms

The management of pulmonary sarcoidosis follows a step-up approach, which aims to suppress excessive immune responses.

### 4.1 First-line therapy: glucocorticoids

Oral prednisone is the first-line choice for patients who are symptomatic or at risk of organ function impairment. Its efficacy stems from the broad suppression of pro-inflammatory cytokine gene transcription, including that of TNF-α and IFN-γ (40). For patients with pulmonary sarcoidosis, the recommended starting dose is 20–40 mg/day for 4–6 weeks (41). The European Respiratory Society (ERS) guideline recommends 20 mg/day as the standard starting dose (42). The SARCORT trial provided key evidence for personalized dosing, confirming that 20 mg/day is as effective as 40 mg/day but with fewer side effects, which supports using a lower starting dose in most patients (43). The specific dose selection should be individualized based on the degree of lung function impairment, symptom severity, and imaging findings, a point also emphasized in expert consensus (41). After an effective response, the dose should be tapered slowly over 6–18 months to a maintenance dose of 5–10 mg/day. If the maintenance dose cannot be reduced below 10 mg/day, a second-line drug should be added (44). The total course of treatment is typically at least 12 months; chronic or relapsing patients may require longer treatment to prevent recurrence (41).

### 4.2 Second-line therapy: immunomodulators

These are primarily used for patients who fail to taper steroids, have an ineffective response, are intolerant, or require long-term treatment. Methotrexate (MTX) is the preferred second-line drug, at a common dose of 10–15 mg/week (42). In a recent published trial, its potential as a first-line agent has been evaluated (45). This study directly compared methotrexate (starting dose 40 mg/week) with prednisone (20 mg/day), finding that the initial high-dose methotrexate regimen did not show superior clinical efficacy and was associated with adverse reactions. However, the same trial protocol, which titrated methotrexate to a target dose, suggests that if MTX is used as first-line treatment, a weekly dose of 15–20 mg should be tolerated for optimal effect (46). For MTX-intolerant patients, Azathioprine (AZA) at 50–200 mg/day has been shown in a retrospective study to be as effective as MTX in its steroid-sparing effect and in preserving FVC, making it a viable alternative (47). Meanwhile, Leflunomide (10–20 mg/day) is a valuable option for patients intolerant to MTX (48). Mycophenolate mofetil represents another alternative, though evidence remains limited (49).

### 4.3 Third-line therapy: biologics

Mainly used for refractory, severe, or progressive disease. Tumor necrosis factor-alpha (TNF-α) therapy targets this core cytokine pivotal to granuloma formation and maintenance. Among these agents, infliximab is the most extensively studied and demonstrates well-established efficacy, administered via a recommended regimen of 5 mg/kg intravenously at weeks 0, 2, and 6, followed by maintenance infusions every 4–8 weeks (50). Adalimumab (40 mg subcutaneously every 1–2 weeks) can be used as an alternative. B-cell targeted therapy represents another approach, wherein rituximab, an anti-CD20 monoclonal antibody, functions by depleting B cells. Although supporting evidence from large randomized controlled trials remains limited, case series have indicated its potential to improve lung function (e.g., FVC) and exercise tolerance (as measured by the 6-min walk test) in certain refractory patients (51).

### 4.4 Anti-fibrotic therapy

For patients presenting with a progressive pulmonary fibrosis phenotype, combination with anti-fibrotic drugs may be considered. A subgroup analysis of the INBUILD trial (Efficacy and Safety of Nintedanib in Patients with Progressive Fibrosing Interstitial Lung Diseases) indicated that nintedanib can slow the decline in lung function in patients with various progressive fibrosing interstitial lung diseases, including sarcoidosis (52, 53). Recent clinical studies show that the presence of circulating CD34+ fibrocytes and EPCs in peripheral blood is related to the severity of pulmonary fibrosis, especially in patients with idiopathic pulmonary fibrosis (IPF) (54). Furthermore, in a study by Heukels et al. (55), fibrocytes constituted 2.6% of total CD45+ cells in IPF lungs, a statistically significant increase compared to control lungs, suggesting fibrocytes as a possible diagnostic marker and therapeutic target.

### 4.5 Emerging and investigational therapies

Emerging therapeutic strategies, including both disease-modifying agents and traditional Chinese medicine (TCM), are currently being evaluated. Studies show nicotine can modulate the immune system by activating the α7 nicotinic acetylcholine receptor (α7nAChR), down-regulating TNF-α, and enhancing Treg function. Clinical studies indicate that transdermal nicotine patches can improve lung function and are well-tolerated, suggesting that targeting the cholinergic anti-inflammatory pathway may be a novel non-immunosuppressive treatment approach (56–58). Meanwhile, Jiawei Yanghe Decoction (JWYHD) has been shown to effectively inhibit Th17 cell differentiation and the production of related cytokines (IL-17A, TNF-α) by up-regulating the expression of nuclear receptors NR1D1/2. Its effect was comparable to prednisolone in animal models (59).

Given the potential role of microbial antigens like *P. acnes* in the pathogenesis, antimicrobial therapy is considered an exploratory treatment strategy. The rationale is based on molecular studies that have detected various mycobacterial genes (e.g., *gyrA*, *ropB*) in sarcoidosis granulomas. The enzymes encoded by these genes are targets for existing antimicrobial drugs (like fluoroquinolones and rifampin), suggesting that targeting these enzymes with antibiotics could be an innovative strategy (60). Clinical studies have shown tetracyclines (e.g., minocycline) are effective for cutaneous sarcoidosis (61). However, the Phase II CLEAR trial (levofloxacin, ethambutol, azithromycin, and rifabutin) for pulmonary sarcoidosis, while reducing the ESAT-6 immune response, failed to improve physiological indicators such as FVC (62). Therefore, although etiology-targeted antimicrobial therapy is theoretically promising, its clinical application still requires more evidence and is an important direction for future research.

## 5 Conclusion and outlook

**FIGURE 2:**
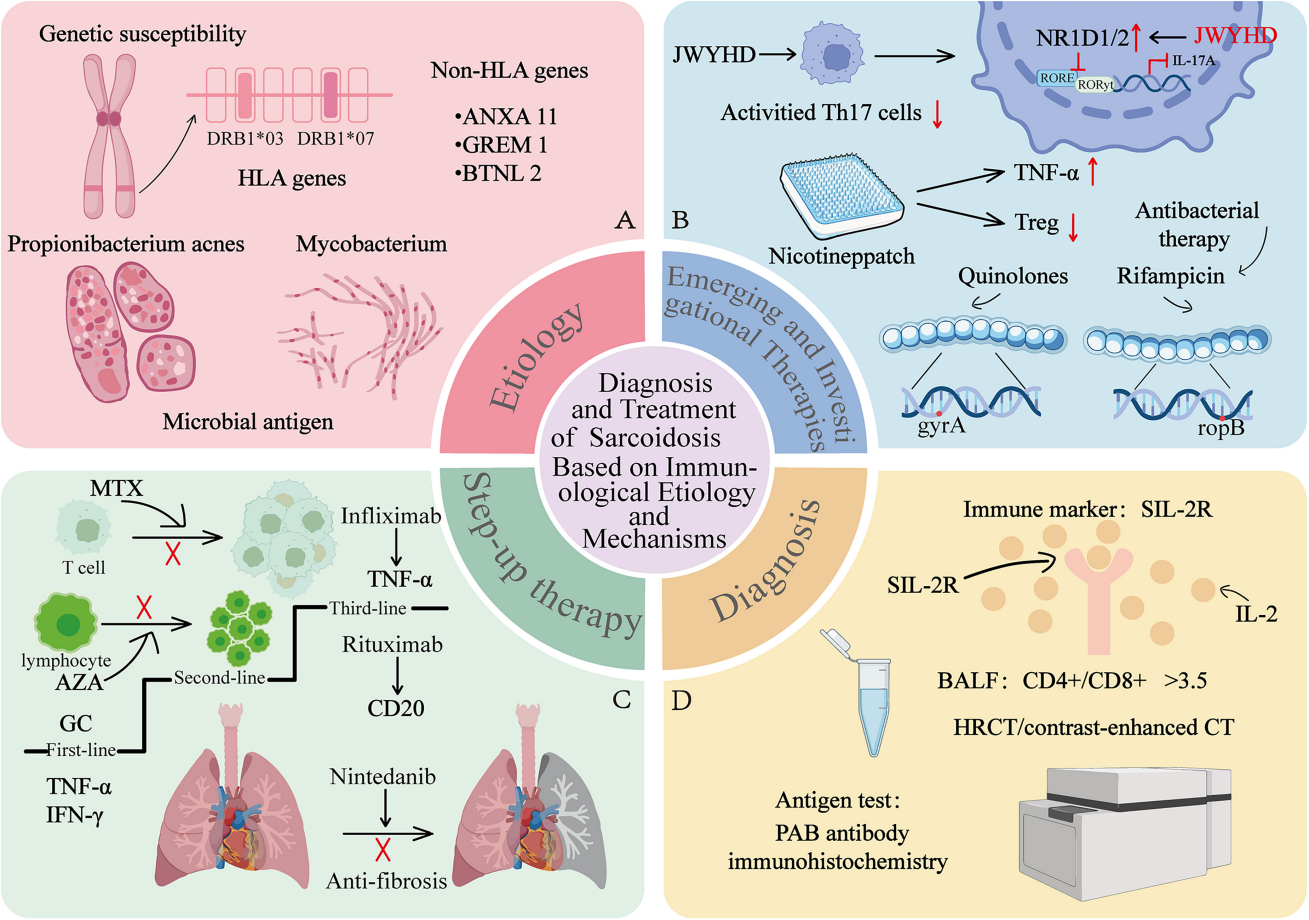
Diagnosis and treatment of sarcoidosis based on immunological etiology and mechanisms. This figure summarizes the etiology, diagnosis, and management of pulmonary sarcoidosis. **(A)** Etiology: Illustrates the interaction between genetic risk factors (e.g., HLA -DRB1*03/07, ANXA11, BTNL2, GREM1) and environmental triggers (*Propionibacterium acnes*, mycobacterial antigens). **(B)** Emerging and Investigational Therapies: Highlights the emerging and investigational therapies including Jiawei Yanghe Decoction (JWYHD), nicotine patches, and antimicrobial therapy (e.g., Quinolones, Rifampicin). **(C)** Step-up therapy: Outlines the stepped treatment approach: GC (1st-line), immunomodulators (MTX, AZA, 2nd-line), biologic agents including infliximab (a TNF-α inhibitor) and rituximab (an anti-CD20 monoclonal antibody, 3rd-line), and anti-fibrotic therapy (Nintedanib). **(D)** Diagnostic Approaches: Details key diagnostic tools: the serum biomarker sIL-2R, an elevated CD4+/CD8+ ratio (>3.5) in BALF, HRCT imaging, and antigen detection via PAB immunohistochemistry. ANXA11, Annexin A11; AZA, Azathioprine; BALF, bronchoalveolar lavage fluid; BTNL2, Butyrophilin-like 2; GC, glucocorticoids; GREM1, Gremlin 1; HLA, Human Leukocyte Antigen; HRCT, high-resolution computed tomography; MTX, methotrexate; PAB, *Propionibacterium acnes* antibody; sIL-2R, soluble Interleukin-2 receptor. This figure was professionally drafted using Adobe Illustrator (version 28.6.0). No AI-based image generation tools were used.

Pulmonary sarcoidosis is an immune-driven granulomatous disease whose diagnosis and management are increasingly informed by immunologic insights. The integration of specific biomarkers-such as serum sIL-2R, BALF CD4+/CD8+ ratio, and functional imaging-enables more precise assessment of disease activity. Management is guided by a step-up strategy, including the use of glucocorticoids, immunomodulators, biologic agents, and antifibrotic therapies, ultimately aiming for individualized care (Figure 2).

Future efforts should focus on novel immune targets (e.g., OX40/OX40L, fibrocytes), refined disease subtyping, and clinical evaluation of emerging approaches, including traditional Chinese medicine and cholinergic modulators. Progress in these areas will directly support the ultimate goal of achieving etiologically targeted intervention.
